# Investigation of the effect of fly ash content on the bonding performance of CFRP-concrete interface in sulfate environment

**DOI:** 10.1038/s41598-022-22537-x

**Published:** 2022-10-19

**Authors:** Shengwei Liu, Qi Li, Jiawei Zhang, Chengyu Bai, Kun Zhao, Gaoming Jin

**Affiliations:** 1 Research Institute, Lanzhou Jiaotong University, Lanzhou, 730070 Gansu China; 2 School of Civil Engineering, Lanzhou Jiaotong University, Lanzhou, 730070 Gansu China; 3 Gansu Jiantou Construction Co. Ltd, Lanzhou, 730070 Gansu China

**Keywords:** Composites, Atomistic models, Mechanical properties

## Abstract

The present study focuses on the investigation of the interfacial bond behavior of carbon fiber-reinforced polymer (CFRP)-concrete under dry–wet sulfate cycles by double-sided shear testing. Besides, the effects of fly ash content on the interfacial failure characteristics, interfacial debonding bearing capacity, CFRP strain distribution, and interfacial shear stress peak were analyzed. The interfacial debonding capacity, maximum CFRP strain, and peak value of interfacial shear stress of the CFRP-concrete interface decreased with increasing erosion time under the sulfate dry–wet cycle's action, according to the sulfate dry–wet cycle test results. The sulfate resistance of the CFRP-concrete interface increased after the addition of fly ash. However, the final decrease amplitude of interfacial debonding capacity, CFRP maximum strain, and maximum interfacial shear stress all reduced as the fly ash content increased. The effective bond length of the interface gradually increased with increasing erosion time; however, the change in fly ash content had little effect on the effective bond length, and the final effective bond length of the samples with different fly ash content was the same. Moreover, the CFRP-concrete interfacial bearing capacity model under the sulfate dry–wet cycle was established by introducing sulfate's comprehensive influence coefficient and considering fly ash content's influence. In conclusion, the comparative analysis of the prediction model and test results revealed that the prediction model could well reflect the degradation law of interfacial debonding bearing capacity with sulfate attack time.

## Introduction

Carbon fiber-reinforced polymer (CFRP) is widely used to support existing reinforced concrete structures by taking its advantages such as a lightweight, not bulky, high strength, easy construction, corrosion resistance, fire resistance, considerable geometric plasticity, and easy tailoring^1–6^. However, most of the CFRP-reinforced structural members of infrastructures are exposed to harsh environmental influences in practical engineering. The bonding performance of the CFRP-concrete interface is directly related to the exposure time to environmental influences, which reduces the bearing capacity of the reinforced elements^7–9^. Therefore, the durability of the CFRP-concrete interface in harsh environments directly affects the long-term mechanical properties of CFRP-reinforced concrete structures. Many scholars^10–14^ have conducted extensive studies on the FRP-concrete interface, resulting in a vast amount of experimental data and helpful conclusions, as well as the development of a more mature interface mechanics model. However, the existing interface mechanics model rarely considers the influence of the erosion environment and cannot accurately express the degradation of FRP-concrete interface performance under an erosion environment.

Sulfate attack is one of the main reasons for the deterioration of mechanical properties of the FRP-concrete interface, according to many studies^15–18^. In one of these studies, Al-Rousan et al. reported that sulfate had a great impact on the interfacial bonding performance of FRP-concrete and that the interfacial bonding performance of FRP-concrete showed significant deterioration after sulfate attack^7^. Zhou et al. studied the effect of sulfate dry–wet cycles on the mechanical properties of CFRP-concrete interface^16^. The test results showed that the interfacial bonding performance decreased significantly under the sulfate dry–wet cycles, indicating that the main factor for the deterioration of the CFRP-concrete interface performance was the deterioration in the mechanical properties of the concrete. The peeling capacity and peak shear stress of the interface declined gradually with the increase in erosion time, according to the experimental results in Reference^15^, and the peeling capacity reduced by around 52% after 150 days of sulfate dry–wet cycle. In addition, it was found that the trend exhibited by the interface peel strength was consistent with the concrete strength based on the change in erosion time. This also indirectly reflects the decrease of concrete mechanical properties under the sulfate attack environment, which is the main reason for the reduction in CFRP-concrete interface bonding performance.

Sulfate attack of concrete is one of the most complex and harmful environmental water physical and chemical erosion, which involves the transmission of sulfate ions in concrete, the reaction between sulfate ions and cement hydration products, and the destruction of concrete structure by the generated expansion products^19–27^. A large number of sulfate ions react with the hydration products of cement, resulting in the decomposition and dissolution significant amount of calcium hydroxide and C-S-H gel material in concrete, which leads reduction in strength properties and cohesiveness of concrete. In addition, the expansion stress generated by these expansion products causes concrete cracking^25–27^. The development of cracks creates a vicious cycle that leads to a sharp decrease in the strength of the concrete, which is sustained by the erosion boundary continually moving towards the interior of the concrete. Although the situation looks underwhelming, some mineral admixtures can improve the resistance of concrete to sulfate attack^28,29^. Among these, fly ash stands out with its advantages such as improving and refining the pore structure of concrete, preventing the penetration of sulfate-containing medium, and slowing the exposure of concrete to sulfate attack^30–33^, thereby effectively increasing the sulfate attack resistance of the FRP-concrete interface. Therefore, investigating the effect of fly ash content on the interfacial bonding performance of CFRP-concrete in a sulfate attack environment is essential for guiding the durability design and assessment of CFRP-reinforced concrete structures.

In this paper, the influence of fly ash content on the bonding properties of CFRP-concrete interface under sulfate dry–wet cycle is studied by double-sided shear test. The mechanical model of CFRP-concrete interface considering the influence of fly ash content under sulfate dry–wet cycle is established.

## Tests

### Test materials and specimen fabrication

The materials used in the study are as follows: Qilianshan grade 42.5 ordinary Portland cement; natural river sand as fine aggregate with a fineness modulus of 3.0; gravel with a maximum particle size of 20 mm as coarse aggregate (the weight ratio of 5–10 mm particle size to 10–20 mm particle size is 1:2); fly ash with good quality class II fly ash; the tap water as mixing water, and UNF-I as the high performance water reducing agent. The indexes of cement and fly ash are shown in Tables 1, 2, 3. According to the different replacement rates of fly ash (0%, 10%, 15%, 20%, 25%), five different mixture design was determined. The mixture proportions of concrete are shown in Table 4.

**Table 1:** Cement performance indicators.

Data type	Standard consistency water consumption (mass fraction)/%	Setting time/min		Fineness (80 μm sieve)/%	Break off strength/MPa		Compressive strength/MPa	
		Initial set	Final set		3 d	28 d	3 d	28 d
GB 175-2007^34^	21–30%	≥ 45	≤ 390	≤ 10	≥ 3.5	≥ 6.5	≥ 17.0	≥ 42.5
observed data	28	140	220	3.0	3.9	8.1	19.7	43.6

**Table 2:** Technical performance indexes of fly ash.

Data type	Fineness/% (0.045 mm)	Water demand ratio/%	The content of SO_3_ (%)	Moisture content (%)	Ignition loss (%)	Quality grade
GB 1596-2005^35^	≤ 25	≤ 105	≤ 3.0	≤ 1.0	≤ 8.0	Level II
observed data	16.4	98	1.22	0.8	6.2	Level II

**Table 3:** Chemical composition of fly ash.

Chemical composition	SiO_2_	Al_2_O_3_	Fe_2_O_3_	CaO	MgO	SO_3_
Fly ash used	48.28%	25.58%	12.34%	3.34%	0.81%	1.22%

**Table 4:** Concrete mix ratio.

W/C	Cement/kg m^−3^	Fly ash/kg m^−3^	Fly ash ratio/%	Water/kg m^-3^	Fine aggregate/kg m^−3^	Coarse aggregate/kg m^−3^	Compressive strength/MPa
0.53	330.0	–	–	175	662	1228	32.8
	297.0	33.0	10	175	662	1228	33.1
	280.5	49.5	15	175	662	1228	30.9
	264.0	66.0	20	175	662	1228	31.3
	247.5	82.5	25	175	662	1228	30.1

**Figure 1:**
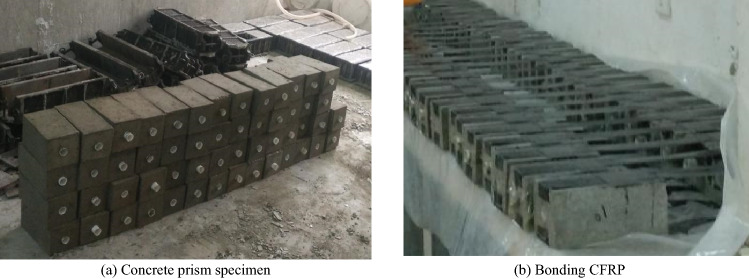
Fabrication of CFRP-concrete double shear specimens.

**Figure 2:**
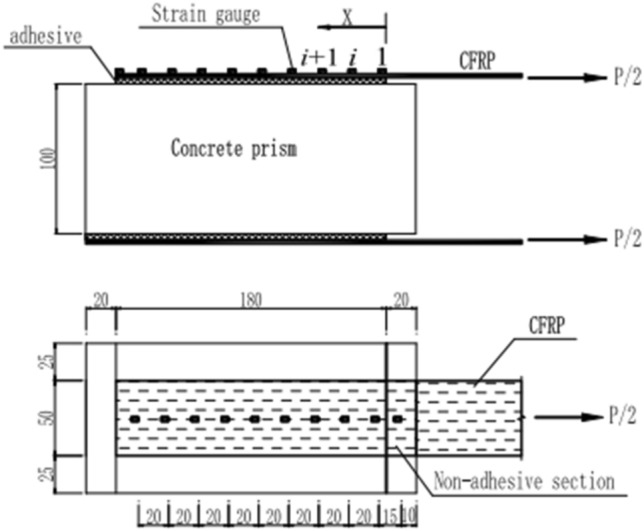
Specimen schematic diagram.

Double-sided shear specimens were used in the experiments. The size of the concrete prism was 100 mm × 100 mm × 220 mm, and 18 prism specimens were prepared for each mixture ratio. The prism specimens are shown in Fig. 1a. At the same time, 18 cubes of 100 mm × 100 mm × 100 mm were produced for compressive strength experiments. After curing for 24 h under ambient conditions, the specimens were removed and kept in a curing room with a temperature of 20 ± 2 °C and relative humidity of 95% for 28 days. Upon completion of the curing process, the CFRP was symmetrically bonded on both sides of the concrete surface. The bond length and width of CFRP were determined as 180 mm and 50 mm, All double shear specimens were not brushed with resin glue within 20 mm on the loading side. The bonding position of CFRP is shown in Fig. 2.

The production process and requirements of the double shear specimen: (1) The concrete specimen is first polished to remove the floating slurry layer and uneven part of the specimen surface at the CFRP paste part until the coarse aggregate is exposed; (2) Cleaning the concrete surface with a blower and then wiping it with anhydrous ethanol; (3) Using the transparent tape to isolate the adhesive area and non-adhesive area, so as to avoid the overflow of resin glue in the process of brushing into the non-adhesive area and affect the test results; (4) Compressing back and forth with rollers in order to eliminate the gaps between the concrete surface and the carbon fiber fabric by the resin-impregnated on the carbon fiber fabric after bonding of CFRP sheets; (5) Curing of the prepared samples at room temperature for 7 days before performing the tests. The field condition of prism specimens bonded with CFRP is shown in Fig. 1b.

### Accelerated aging process conditions

The dry–wet cycle system was carried out according to the sulfate resistance test standard of GB/T50082-2009^36^. The test was carried out with 10% Na_2_SO_4_ solution. The dry–wet cycle process was as follows: Soaking in sulfate solution for 12 h, then discharging the solution, drying for 2 h, heating the test box, drying for 8 h at 40 °C, and finally shutting down the heating system for natural cooling for 2 h. After one cycle is complete, the sample is placed in solution for the next 24-h cycle. The temperature of the test box is maintained at 40 ± 2 °C during drying operation due to the softening temperature of the adhesive resin is 45–80 °C^17,37,38^. The solution is renewed every other month to maintain the constant concentration. A double set of shear specimens and a cube specimen were produced, with three specimens in each group, at five mixing ratios, to be tested every 30 days.

In order to facilitate the analysis of test results, all specimens were grouped. In this grouping, while DW represents the sulfate dry–wet cycle environmental effect; F0, F10, F15, F20 and F25 represent concrete fly ash content of 0%, 10%, 15%, 20% and 25%, respectively. Sulfate attack time was directly marked by digital. For example, DW-F10-60 represents a group of specimens with 10% fly ash content and 60d erosion time under sulfate dry–wet cycle. The specific specimen grouping is shown in Table 5.

**Table 5:** Summary of test results.

Specimen group number	Fly ash content (%)	The erosion of time (d)	Maximum shear stress/MPa	Peeling capacity/kN	Destruction mode
DW-F0-0	0	0	5.64	20.2	I
DW-F10-0	10	0	5.65	19.6	2I + II
DW-F15-0	15	0	5.61	18.9	I
DW-F20-0	20	0	5.59	20.4	I
DW-F25-0	25	0	5.63	19.5	I
DW-F0-30	0	30	5.74	20.5	2I + II
DW-F10-30	10	30	5.68	20.3	2I + II
DW-F15-30	15	30	5.72	19.4	I
DW-F20-30	20	30	5.67	20.3	I
DW-F25-30	25	30	5.71	20.1	I
DW-F0-60	0	60	5.28	18.5	II
DW-F10-60	10	60	5.37	18.6	II
DW-F15-60	15	60	5.48	18.5	I + 2II
DW-F20-60	20	60	5.57	20.8	2I + II
DW-F25-60	25	60	5.56	19.6	I + 2II
DW-F0-90	0	90	4.67	16.1	II
DW-F10-90	10	90	4.73	16.5	II
DW-F15-90	15	90	4.84	16.7	II
DW-F20-90	20	90	5.04	18.1	II
DW-F25-90	25	90	4.96	17.7	II
DW-F0-120	0	120	3.92	12.8	II
DW-F10-120	10	120	3.96	13.1	II
DW-F15-120	15	120	4.13	13.6	II
DW-F20-120	20	120	4.45	14.8	II
DW-F25-120	25	120	4.43	14.3	II
DW-F0-150	0	150	3.02	9.7	II
DW-F10-150	10	150	3.17	9.6	II
DW-F15-150	15	150	3.43	10.3	II
DW-F20-150	20	150	3.65	11.9	II
DW-F25-150	25	150	3.69	11.5	II

### Loading and measurements

**Figure 3:**
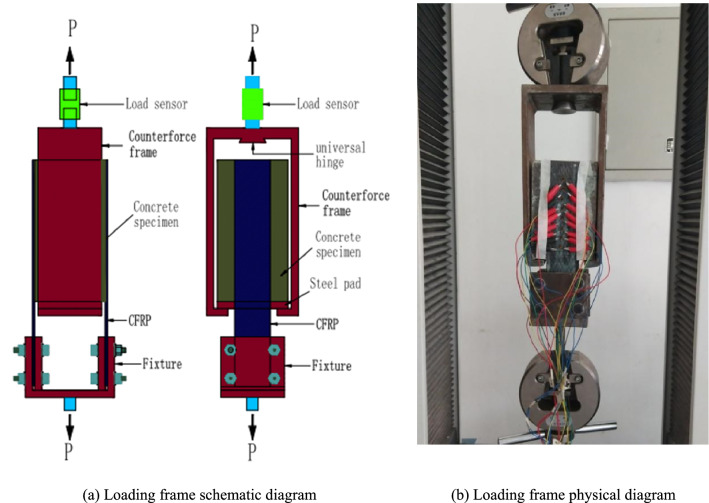
Loading device schematic diagram.

The electronic universal testing machine for the CFRP-concrete double shear test is shown in Fig. 2. The loading process was controlled by displacement, and the loading rate was selected as 0.2 mm/min. Before applying tensile force to the sample, a pre-load of approximately 0.4 kN was applied to check that the fixture, tensile machine, load sensor, and strain-collection system were working properly. After checking the test system, the tensile test of the double shear specimens was performed and the test process, specimen failure mode, and test data were recorded. The loading device is shown in Fig. 3.

## Test results

### Concrete strength

The residual strength ratio of concrete is utilized to more clearly articulate the attenuation law of concrete strength with erosion time under the sulfate dry–wet cycle. The expression of residual strength ratio of concrete is as follows:

1 $$ R = \frac{{_{{f_{cT} }} }}{{_{{f_{co} }} }} $$

where $R$ is the residual strength ratio of concrete, ${\text{f}}_{c0}$ is the compressive strength of concrete under standard curing age (28 days), and $f_{cT}$ is the compressive strength of concrete under each erosion age.

**Figure 4:**
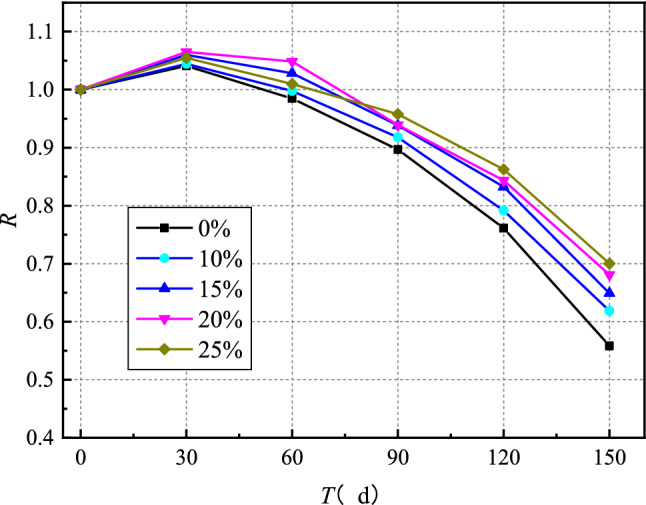
Time-varying curve of concrete compressive strength.

The variation of compressive strength of concrete with erosion time under sulfate dry–wet cycle is presented in Fig. 4. The variation of compressive strength of concrete with different fly ash content with erosion time is basically similar, which can be divided into two stages: a slight increase in concrete strength in the early stage of erosion and a decrease in concrete strength in the late stage of erosion. There are two main reasons for the slight increase in the compressive strength of concrete in the first stage. The first one is that the hydration of cement is insufficient during the 28 days curing period, and the hydration of cement continues to take place in the matrix, resulting in the improvement of the strength of concrete. The second reason is that sulfate ions react with the hydration products of the cement in the concrete to form expansion products such as gypsum and ettringite, which fill the pores of the concrete. Under the combined action of these two factors, the concrete becomes denser, and the mechanical properties show a small increment. In the second stage, a large number of sulfate ions react with the hydration products of cement, resulting in a significant amount of calcium hydroxide and C-S-H gel decomposing and dissolving in concrete, which leads to a decrement in the cohesive properties and strength of concrete^25,26^. Furthermore, the expansion stress caused by these expansion products leads concrete to crack. The development of cracks leads the erosion boundary to advance continually to the interior of the concrete, resulting in a vicious cycle in which the concrete's strength is dramatically reduced^25–27^. According to the results, after 150 days of erosion, the strength of concrete without fly ash decreased by 41.2%.

As shown in Fig. 4, with the increase of fly ash content, the time of curve inflection point became more prolonged, and the ultimate strength decreased. The strength of the specimens without fly ash began to decrease after 30 days of erosion. In the case where the fly ash content was 20% and 25%, the strength began to decline significantly after 60 days of erosion. After 150 days of erosion, the strength of concrete with 10%, 15%, 20% and 25% fly ash and without fly ash decreased by 41.2%, 38.1%, 36.1%, 31% and 32.1%, respectively. Fly ash can be chemically stable and physically refine the pore structure of concrete^30,31^. In addition, the substitution of fly ash to cement reduces the ${C}_{3}A$ from the cement, in other words, the raw materials for reaction with sulfate ions. Besides, fly ash also performs secondary hydration with $\mathrm{Ca}{(\mathrm{OH})}_{2}$ in concrete, which reduces the $\mathrm{Ca}{(\mathrm{OH})}_{2}$ content and makes the matrix denser, creating a relatively stable C-S-H. Usually, fly ash's fineness, and particle size are smaller than cement clinker, which increases the density of concrete to a certain extent and improves the sulfate corrosion resistance of concrete^31–33^. It can also be seen from Fig. 4 that increasing the fly ash content from 20 to 25% did not cause an apparent variation for compressive strength with erosion time was relatively close, indicating that after a threshold for fly ash content, the sulfate corrosion resistance of concrete did not increase. The reason behind this is that when the fly ash content exceeds a threshold, a large pore solution area is formed in the concrete during the sulfate dry–wet cycle and damages the concrete due to the refinement effect of the fly ash on the pore size of the concrete^39^.

### Interface failure patterns

**Figure 5:**
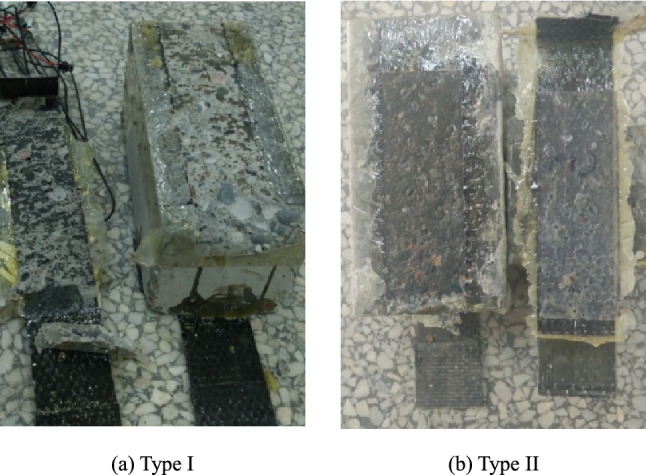
Typical damage patterns of specimens.

After sulfate dry–wet cycles, the specimen's failure mode changed significantly from the failure of the concrete layer without erosion to the failure of the interface between colloid and concrete. The specimen's failure surface occurs in the concrete layer underneath the adhesive layer without sulfate attack or with a short erosion period. During failure, a large number of pulled concrete debris and particles adhered to the CFRP, and the concrete surface was in a rough state. At the loading end, a triangular shear zone with an angle of about 45 degrees with the CFRP axis appeared, called type I failure. The failure surface occurred at the concrete surface or the bonding interface for the specimens exposed to a long sulfate attack. The CFRP sheet was no longer bonded with concrete debris but only a small amount of concrete particles and the triangular shear zone at the loading end could not be observed, as identified as a type II fault. Typical failure characteristics of specimens are shown in Fig. 5.

The mechanical properties of CFRP sheets showed a slight decrease under sulfate dry–wet cycles. However, because the strength of CFRP and bond resin was much higher than that of concrete, the decrease in the performance of CFRP and bond resin had little effect on the degradation of the bond performance of the CFRP-concrete interface^15^. Therefore, the change in the failure mode of the CFRP-concrete interface was mainly caused due to the degradation of concrete and the deterioration of the contact surface. Based on the analysis in “Concrete strength” section, it can be seen that the strength of concrete tends to decrease gradually with increasing sulfate dry–wet cycle time, resulting in decreased CFRP-concrete interfacial bonding performance. The addition of fly ash increased the sulfate resistance of the concrete, thus improving the corrosion resistance of the interface. Moreover, as seen in Table 5, the time required for the failure mode of the sample with high fly ash content to change from type I failure to type II failure was longer. For example, the sample with 25% fly ash content also had type I failure after 60 days of sulfate dry–wet cycle. All the findings revealed that the incorporation of an appropriate amount of fly ash into concrete can effectively improve the sulfate resistance of the CFRP-concrete interface.

### Interface stripping capacity

With increasing sulfate attack time, the interface bearing capacity of CFRP-concrete will deteriorate. To eliminate the problem that the test results due to the heterogeneity of the concrete show variations, the effect of the fly ash content and the drying-wetting cycle time of the sulfate on the interfacial bearing capacity of CFRP-concrete are examined as follows: While the erosion time T was taken as the abscissa, the ratio of the interfacial abrasion bearing capacity obtained with different erosion durations to the interfacial wear bearing capacity of the same type samples at room temperature, (${\mathrm{P}}_{\mathrm{u},\mathrm{T}}/{\mathrm{P}}_{\mathrm{u},0}$), was taken as the ordinate.

**Figure 6:**
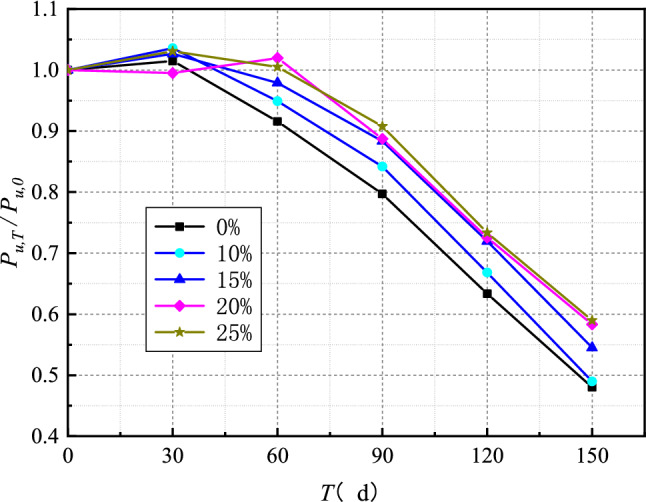
Effect of fly ash content on stripping bearing capacity.

The variation curve of stripping bearing capacity of specimens with various fly ash parameters during sulfate dry–wet cycles is shown in Fig. 6. It is essentially the same, as shown in the Figure. The peel strength of the interface normally shows a downward trend as the sulfate erosion duration increases, but there is a slight rise in the early stage of erosion, which is due to the partial hydration of cement in concrete and the increase of strength in the later stage. Through the comparison between Figs. 4 and 6, it can be seen that the time-varying curves of interfacial debonding bearing capacity and concrete strength are quite similar, indicating that the decline in concrete mechanical characteristics is the primary cause of the drop in interfacial bearing capacity. According to the curve in Fig. 6, the time necessary for the interface debonding bearing capacity to begin to drop gradually grows longer as the concrete fly ash content increases and the rate of decline gradually slows. The interfacial debonding bearing capacity retention rates of specimens without fly ash and specimens with 25% fly ash were 48% and 59%, respectively, after 150 days of sulfate dry–wet cycle. It reveals that the sulfate corrosion resistance of the CFRP-concrete interface is better when the concrete fly ash content is relatively high.

### Strain distribution

**Figure 7:**
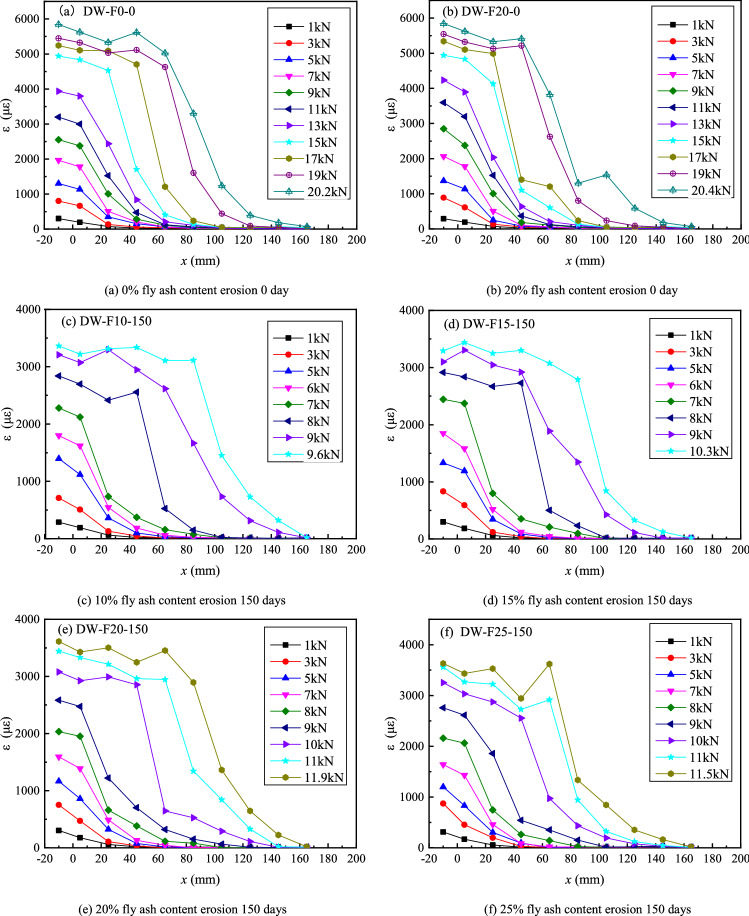
FRP strain distribution of various specimens under different loading levels.

The distribution of CFRP stresses of different specimens in the bond length direction can be obtained using strain gauges pasted on the CFRP surface when the load varies. CFRP strain varies with distance from the loading end x at different load levels, as shown in Fig. 7. The development of CFRP strain in all double shear specimens is the same throughout the loading process, and the CFRP strain gradually stretches from the loading end to the free end. The interface at the loading end begins to peel off as the load approaches the stripping load, and the CFRP strain at the loading end reaches its maximum. The peak strain of CFRP advances to the free end constantly as the load increases, and the strain curve appears as approximate parallel section near the side of the loading end, and the whole curve is nearly “S” shape. The interface is destroyed with a loud noise when the peeling develops to the free end. However, until all specimens are destroyed, the strain value at a distance near the free end is still very tiny. At the same time, the ultimate strain of CFRP gradually reduces as the sulfate dry–wet cycle time increases.

It is obtained by comparing the strain distribution of CFRP with the different fly ash content in Fig. 7 that the rate of decrease of the ultimate strain of the CFRP slows down significantly with the increase of the fly ash content. For example, after 150 days of erosion, the ultimate strain of CFRP of specimens without fly ash is between 3000 and 3500; when the fly ash content is 20%, the ultimate strain of CFRP is between 3500 and 4000.

### Interface shear stress distribution

Strain gauges on the surface of CFRP can be used to determine the strain distribution at each position. The corresponding local bond shear stress can be calculated using the following difference Eq. (2) based on the strain distribution of each point on the surface of CFRP:

2 $$ \tau_{f,i} = \frac{{E_{f} t_{f} d_{{\varepsilon_{f} }} }}{{d_{x} }} = \frac{{\left( {\varepsilon_{f,i} - \varepsilon_{f,i - 1} } \right)t_{f} E_{f} }}{{\Delta l_{b,i} }} $$

where $\tau_{f,i}$ is the average bond shear stress at $i - 1$ and $i$ points; $\varepsilon_{f,i}$ is the strain value at $i$ points; $\Delta l_{b,i}$ is the distance between $i - 1$ and $i$; $E_{f}$ and $t_{f}$ are the elastic modulus and thickness of CFRP, respectively.

**Figure 8:**
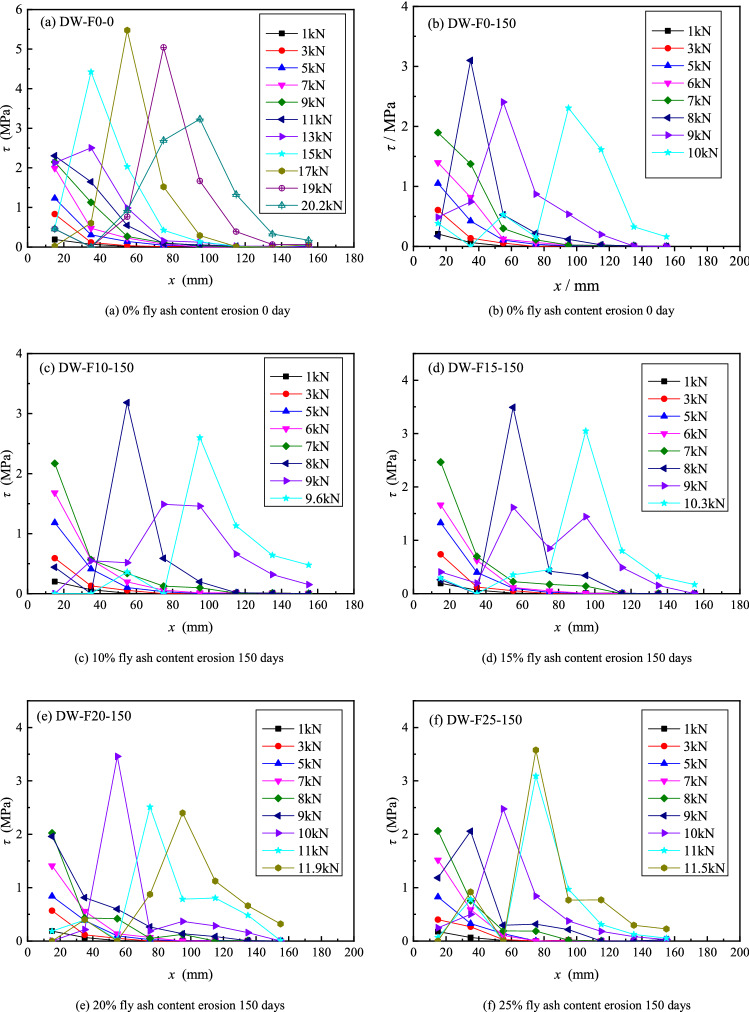
Shear stress distribution of various specimens under different loading levels.

Figure 8 depicts the interfacial shear stress distribution along the bond direction of CFRP under different load conditions. As showing in Fig. 8. With the increase of load, the interface shear stress at the loading end increases and transfers to the free end. When the load is close to the peeling load, the interface shear stress at the loading end begins to decrease, and the interface peak shear stress continues to move to the free end until the interface peeling failure, but the interface shear stress near the free end is still very small when the specimen is destroyed, it can be seen from Fig. 8 that the maximum interfacial shear stress is almost not at the first point of the loading end. When the interfacial shear stress curves of different types of samples are compared, it is seen that the maximum interface shear stress gradually decreases with the increase of the erosion time, while the decrease in the interface shear stress decreases significantly as the fly ash content increases.

**Figure 9:**
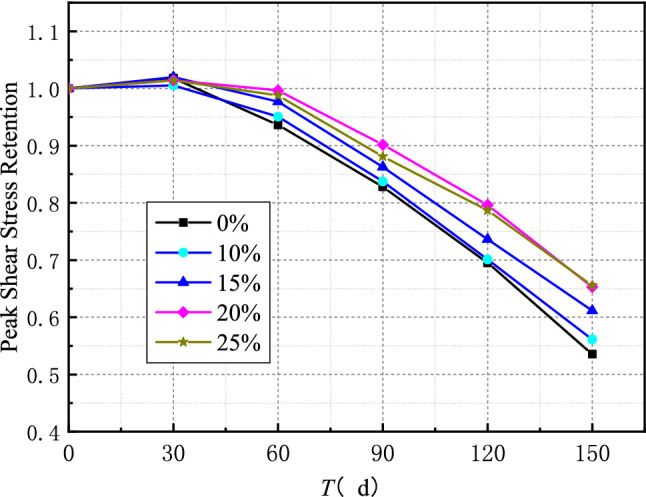
Time-varying curve of peak shear stress.

The retention rate of the peak shear stress of the interface (the ratio of the peak shear stress of different types of specimens corresponding to each erosion time to the peak shear stress of the interface without erosion) is used to represent the change of the interface shear stress with erosion time in order to more intuitively express the influence of fly ash content and erosion time on the maximum shear stress of the interface. Figure 9 shows the relationship between interface peak shear stress and erosion time. It can be seen from Fig. 9 that the interface peak shear stress decreases with the increase of erosion time; also, the decrease of interface peak shear stress is smaller with the increase of fly ash content. The retention rates of peak shear stress at the interface of specimens without fly ash and specimens with 25% fly ash after 150 days of sulfate dry–wet cycle were 53% and 65%, respectively.

### Effective bond length

The bond strength between CFRP and concrete does not always increase with the increase of bond length. Many investigations have revealed that at the CFRP-concrete interface, there is an effective bond length, $L_{{\text{e}}}$. The effective bond length was defined by Yuan et al. as the bond length corresponding to 97% of the total shear stress within the bond length range^40^; however, measuring the effective bond length directly when reaching the ultimate load is difficult. Nakaba et al. recommended that the effective bond length is taken as the distance between the two points on both sides of the peak shear stress distribution (the coordinate point corresponding to the peak shear stress of 10%)^41^, that is, the effective stress transfer length. Because the stress transfer length is consistent with the length corresponding to the inclined section of the strain curve, the effective bond length is defined as the bond length corresponding to the inclined section of the strain curve.

**Figure 10:**
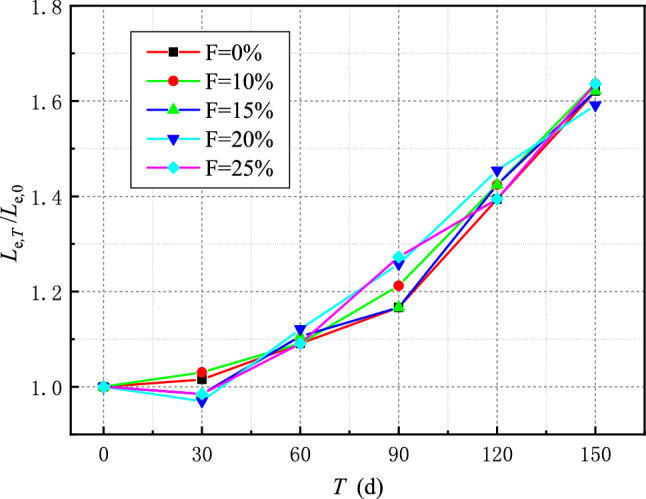
Time-varying curve of effective bond length at the interface.

The presence of interfacial friction causes the CFRP strain to increase somewhat following interfacial debonding, as seen from the specimen's strain curve analysis. As a result, the effective bond length is calculated as the length of an inclined section between two points on the fitting curve with maximum strain values of 2% and 98%. According to the strain curve analysis, the effective bond length of the FRP interface at room temperature is 60–70 mm, and the fly ash content has little effect on the effective bond length. The effective bond length of FRP will grow as the sulfate dry–wet cycle time increases. The effective bond length of specimens with different fly ash yields at room temperature was normalized to minimize the heterogeneity of concrete and the discreteness of test results induced by the difference in specimen production. The change in effective bond length of the interface between distinct specimens throughout dry and wet sulfate cycles is depicted in Fig. 10.

The effective bond length of the interface increases as the erosion duration increases, as shown in Fig. 10. However, the content of fly ash has minimal effect on the change of effective bond length with erosion time. Therefore, the same function can reflect the variation of effective bond length with erosion time. The sulfate influence coefficient $\eta_{L}$ was introduced to establish the effective bond length calculation equation when considering the sulfate dry–wet cycle erosion time:

3 $$ L_{e,T} = \eta_{L} L_{e,0} = 0.933\eta_{L} \sqrt {\frac{{E_{f} t_{f} }}{{\sqrt {f_{c} } }}} $$

where $L_{e,T}$ is the effective bond length when the corrosion time is T, $L_{e,0}$ is the effective bond length when the interface is not corroded.

**Figure 11:**
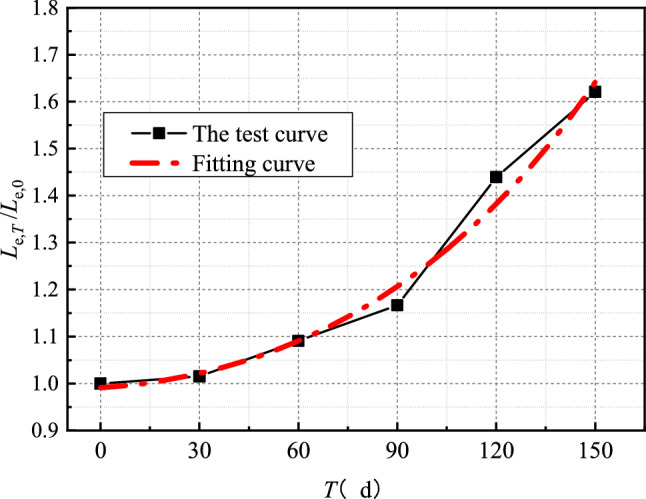
Time-varying curve of effective bond length at the interface.

Figure 11 is the distribution map of the effective bond length of the interface at different erosion times. By fitting the test curve of Fig. 11, the expression of effective bond length influence coefficient *η*_L_ under sulfate dry–wet cycle is obtained

4 $$ \eta_{L} = e^{{ - 8.85 \times 10^{ - 3} + 4.19 \times 10^{ - 4} T + 1.96 \times 10^{ - 6} T^{2} }} $$

## Interface bearing capacity model

Domestic and foreign scholars have established some bearing capacity models through a large number of experimental research and theoretical analyzes, such as: Hiroyuki and Wu model^42^, Tanaka model^43^, Chaallal et al. model^44^, Sato model^45^, Izumo model^46^, Iso model^46^, Neubauer and Rostasy model^47^, Khalifa model^48^, Chen and Teng model^49^, Lu Xinzheng model^50^. However, the existing interfacial bearing capacity models are mainly proposed without taking environmental factors into account. The analysis of the existing interfacial bearing capacity models showed that the interfacial bearing capacity is closely related to the stiffness of fiber sheets and the strength of concrete. However, after sulfate dry–wet cycles, the mechanical properties of the constituent materials will deteriorate with the extension of erosion time. Therefore, the existing interfacial bearing capacity models cannot accurately reflect the loss of interfacial bearing capacity after sulfate erosion. This paper presents an interfacial bearing capacity model for sulfate erosion based on the existing interfacial bearing capacity model by introducing the comprehensive effect coefficient $\eta_{s}$ of sulfate:

5 $$ P_{u,T} = A\eta_{S} \beta_{l} \beta_{w} b_{f} \sqrt {E_{f} t_{f} \sqrt {f_{c0} } } $$

where $P_{u,T}$ is the interface stripping bearing capacity of erosion time $T$; $\beta_{l}$ is the bond length influence coefficient; $\beta_{w}$ is width influence coefficient; $A$ is constant coefficient.

The constant coefficient $A = 0.436$ can be obtained by substituting the interfacial debonding bearing capacity obtained without fly ash at room temperature into Eq. (5). The impact of fly ash content on interfacial bearing capacity is investigated in the following study based on the test results under these conditions.

### Combined sulfate impact factor

**Figure 12:**
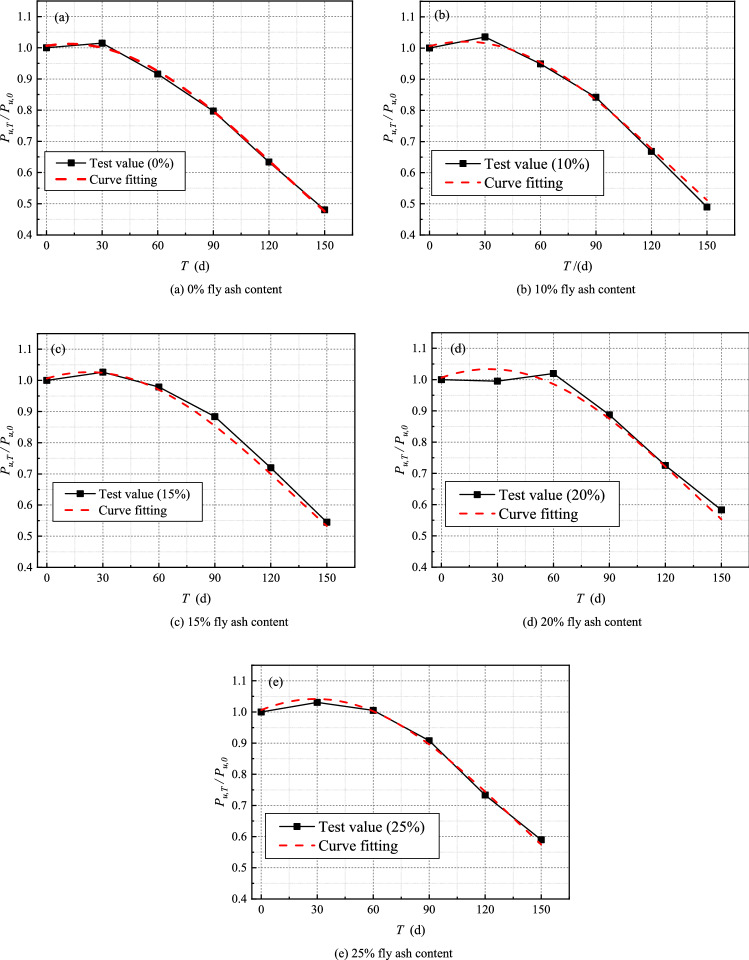
Time-varying curve of stripping bearing capacity for different fly ash content.

As the sulfate dry–wet cycle time increases, the interfacial bearing capacity of CFRP-concrete decreases. The effect of erosion time on the interfacial bearing capacity of specimens without fly ash is investigated using the change in interfacial debonding bearing capacity of specimens without fly ash with sulfate dry–wet cycle time. Similarly, the interfacial debonding bearing capacity obtained at different erosion durations is normalized with the average value of the specimen at room temperature to eliminate the large discreteness of test findings caused by concrete heterogeneity and differences in specimen preparation. The variation trend of interface debonding bearing capacity with erosion time can be calculated using erosion time $T$ as abscissa and ${{P_{u,T} } \mathord{\left/ {\vphantom {{P_{u,T} } {P_{u,0} }}} \right. \kern-\nulldelimiterspace} {P_{u,0} }}$ as ordinate, as shown in Fig. 12a. To obtain the expression of the comprehensive influence coefficient of sulfate under the dry–wet cycle of sulfate, Eq. (5) is used to fit the data in Fig. 12a:

6 $$ \eta_{s} \left( T \right) = e^{{0.0062 + 9.975 \times 10^{ - 4} T - 3.997 \times 10^{ - 5} T^{2} }} $$

The bonding performance of the CFRP-concrete interface under sulfate assault has improved to some extent after adding fly ash to concrete.

The interface bearing capacity diminishes as the amount of fly ash in the mixture increases. As a result, the comprehensive effect coefficient of sulfate will also change as the fly ash content changes. The complete effect coefficient of sulfate in a sulfate environment must be rectified. When Eq. (5) is used to calculate the bearing capacity, the comprehensive influence coefficient of the sulfate $\eta_{s}$ in sulfate environment must be corrected. Equation (6) can be expressed as:

7 $$ \eta_{s} \left( T \right) = k_{F} e^{{0.0062 + 9.975 \times 10^{ - 4} T - 3.997 \times 10^{ - 5} T^{2} }} $$

where $k_{F}$ is the correction coefficient of fly ash content.

Figure 12 shows the change in interface debonding bearing capacity with erosion time when fly ash content is 10%, 15%, 20%, and 25%, respectively. The relationship between the correction factor $k_{F}$ of fly ash content and erosion time T can be obtained by fitting the data in the diagram with Eq. (7). The correction coefficient $k_{F}$ expression of fly ash content can be calculated by regression calculation:

8 $$ k_{F} = 1 + \frac{0.93T}{{150}}F^{1.08} $$

where $F$ is the content of fly ash.

The expression of the complete effect coefficient $\eta_{s}$ considering fly ash content may be found by substituting Eqs. (8) into (7):

9 $$ \begin{aligned} \eta_{s} \left( T \right) & = k_{F} e^{{0.0062 + 9.975 \times 10^{ - 4} T - 3.997 \times 10^{ - 5} T^{2} }} \\ & = \left( {1 + \frac{0.93T}{{150}}F^{1.08} } \right)e^{{0.0062 + 9.975 \times 10^{ - 4} T - 3.997 \times 10^{ - 5} T^{2} }} \\ \end{aligned} $$

Substituting Eqs. (9) into (5), the bearing capacity model of CFRP-concrete interface under sulfate dry–wet cycle is obtained.

### Comparative analysis of prediction model and test results

**Figure 13:**
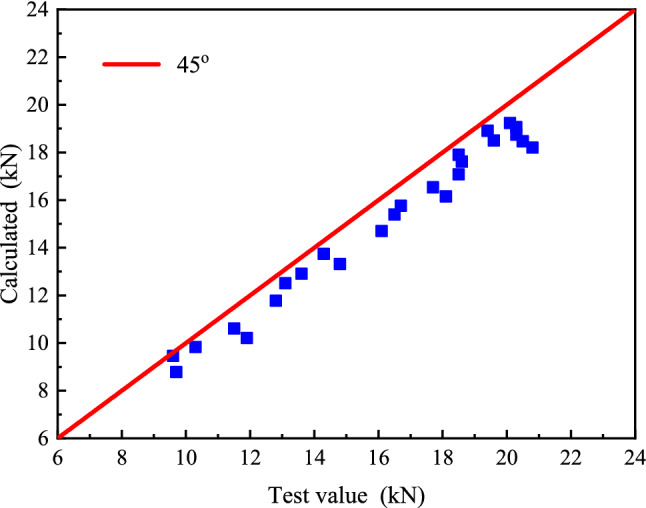
Comparison between model predictions and test data.

The interface bearing capacity under different working conditions was calculated according to the Eqs. (5) and (9). A comparison was made between the calculated value and the experimental value by taking the interfacial stripping bearing capacity obtained from the experiment as the abscissa and the calculated value of the model as the ordinate (Fig. 13). As can be seen in the diagram, the data points of the calculation value and the experimental value of the debonding bearing capacity of the CFRP-concrete interface under the influence of the sulfate dry–wet cycle are distributed below the 45° line. The fundamental reason for this is that the interfacial debonding bearing capacity increases slightly due to the friction and bite force generated at the debonding surface. However, when the bond length surpasses the effective bond length, the growing influence of friction and mechanical bite force on the interfacial debonding bearing capacity is not considered in the prediction model. Hence, the experimental value is greater than the model's predicted value.

## Conclusions

In the current study, the effect of fly ash content on the bonding performance of the CFRP-concrete interface was studied by the sulfate dry–wet cycle accelerated erosion test. The specific conclusions are as follows:

1. After the sulfate dry–wet cycles, the interfacial failure gradually changed from the concrete layer below the adhesive layer to the damage at the interface between the adhesive layer and the concrete, with increasing erosion time. However, the addition of fly ash improved the sulfate resistance of concrete, thus improving the corrosion resistance of the interface. With the increase of fly ash content, the failure mode of the specimen changed from type I failure to type II failure for a prolonged duration.
2. The decrease in the mechanical properties of concrete was the main reason for reducing CFRP-concrete interface bearing capacity. With the increase of erosion time, the interfacial bond separation carrying capacity decreased; however, with the increase of fly ash content, the time required for the interfacial bond separation carrying capacity to begin to decrease gradually was prolonged, and the final decrease gradually decreased.
3. Under the sulfate dry–wet cycle, the maximum strain value of CFRP and the peak value of interfacial shear stress gradually reduced as erosion duration increased. However, the sulfate resistance of the CFRP-concrete interface improved after the addition of fly ash, and the final state of maximum strain and interfacial shear stress of CFRP gradually decreased with the increase of fly ash content.
4. With the increase of sulfate dry–wet cycle time, the effective bond length of the interface gradually increased. On the other hand, the impact of the fly ash content on the variation of the effective bond length was not notable. The final effective bond length of the samples with different fly ash content was detected to be basically the same.
5. The interface bearing capacity model of CFRP-concrete under the dry–wet cycle of sulfate was established by introducing the comprehensive influence coefficient of sulfate and considering the influence of fly ash content. Through the comparative analysis of the prediction model and the experimental results, it has been revealed that the prediction model can well reflect the degradation law of interface stripping bearing capacity with sulfate attack time.

## Data Availability

The datasets used and/or analysed during the current study available from the corresponding author on reasonable request.
